# Supplementary figures and images for: Sepsis risk in diabetic patients with urinary tract infection
Source: PLoS One. 2024 May 21;19(5):e0303557. doi: 10.1371/journal.pone.0303557 (PMC11108167; doi:10.1371/journal.pone.0303557)

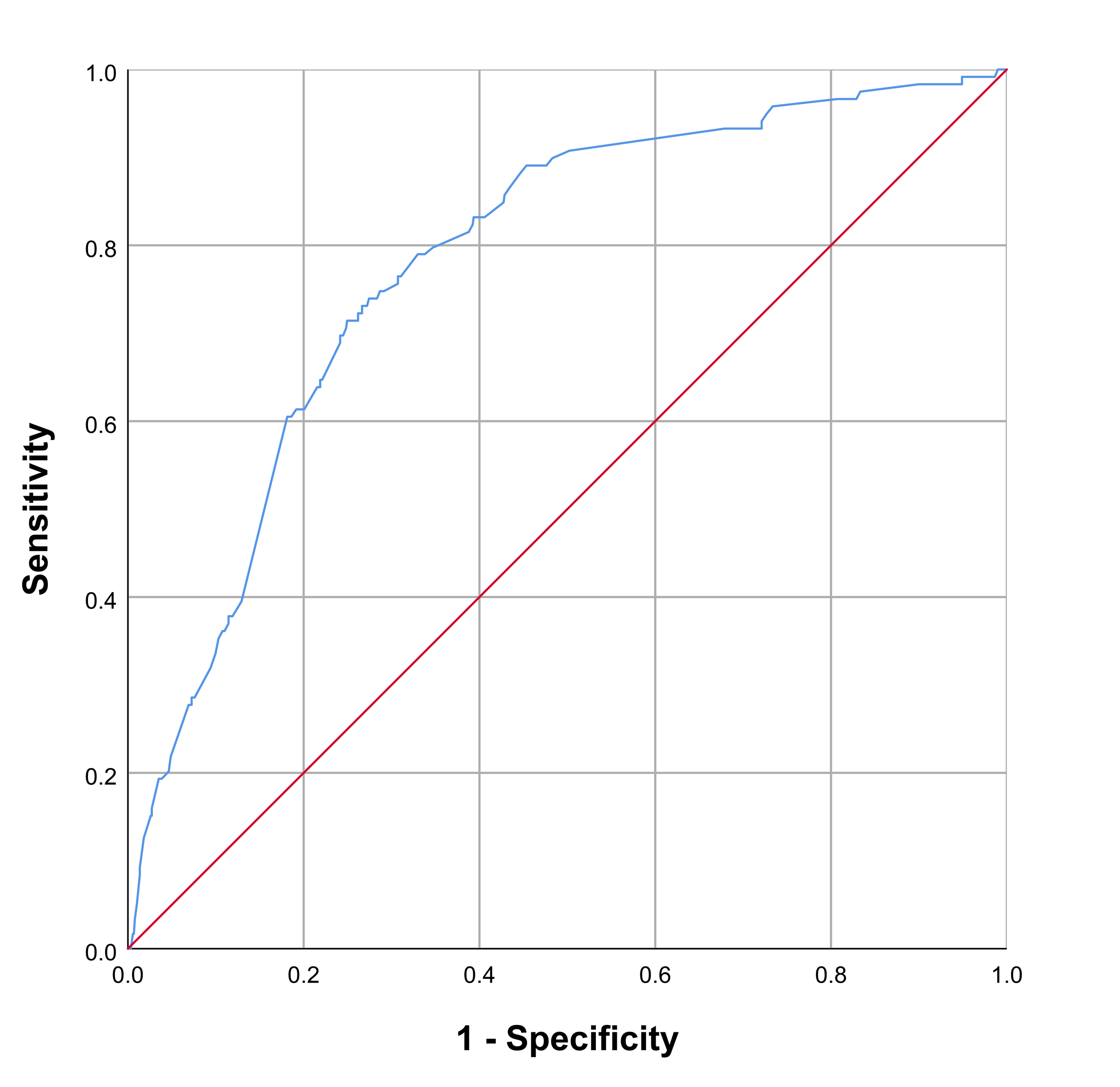

Supplement: S1 Fig — (TIF) [file pone.0303557.s001.tif]
