# Supplementary material for: Sepsis risk in diabetic patients with urinary tract infection
Source: PLoS One. 2024 May 21;19(5):e0303557. doi: 10.1371/journal.pone.0303557 (PMC11108167; doi:10.1371/journal.pone.0303557)
Supplement: S1 Table — (DOCX) [file pone.0303557.s002.docx]

**Supplementary table 1**. The cut-off values of variables related to sepsis.

| Index | AUC (95% confidence interval) | Cut-off | Specificity (%) | Sensitivity (%) |
| --- | --- | --- | --- | --- |
| ALB | 0.782 (0.750-0.814) | 34.35 | 0.709 | 0.764 |
| CRP | 0.787 (0.756-0.817) | 55.84 | 0.729 | 0.715 |
| PCT | 0.849 (0.822-0.877) | 1.015 | 0.847 | 0.717 |
| HbA1c | 0.595 (0.553-0.637) | 8.35 | 0.713 | 0.450 |
| WBC | 0.731 (0.697-0.765) | 8.485 | 0.599 | 0.786 |

ALB, albumin; CRP, C-reactive protein; PCT, procalcitonin; HbA1c, glycated haemoglobin; WBC, white blood cell count
