# Supplementary material for: Sepsis risk in diabetic patients with urinary tract infection
Source: PLoS One. 2024 May 21;19(5):e0303557. doi: 10.1371/journal.pone.0303557 (PMC11108167; doi:10.1371/journal.pone.0303557)
Supplement: S2 Table — (DOCX) [file pone.0303557.s003.docx]

**Supplementary Table 2.** The risk factors related to sepsis excluding inflammatory indices (CRP, WBC).

| Index | β | SB | Walsχ2 | P | Exp(B) | Exp(B) 95% CI |
| --- | --- | --- | --- | --- | --- | --- |
| ALB | 1.964 | 0.221 | 78.857 | 0.000 | 7.127 | 4.62-10.994 |
| HbA1c >8.35% | 0.474 | 0.212 | 4.975 | 0.026 | 1.606 | 1.059-2.434 |

ALB, albumin; HbA1c, glycated haemoglobin.
